# Supplementary material for: Near millimolar concentration of nucleosomes in mitotic chromosomes from late prometaphase into anaphase
Source: J Cell Biol. 2024 Aug 26;223(11):e202403165. doi: 10.1083/jcb.202403165 (PMC11346515; doi:10.1083/jcb.202403165)
Supplement: Table S1 — shows chromosome measurements in different mitotic stages. [file JCB_202403165_TableS1.docx]

Table S1. Chromosome measurements in different mitotic stages.

|  | Total volume | Mb/μm^3^ | Mb/μm  (Chromosome – Chr  Chromatid – Chd) | Chromatid width | Chromosome width | Spindle pole separation |
| --- | --- | --- | --- | --- | --- | --- |
| Prometa 1 | 256 μm^3^ |  |  |  |  | 6.71 μm |
| Prometa 2 | 209 μm^3^ |  |  |  |  | 9.15 μm |
| Prometa 3 | 184 μm^3^ |  |  |  |  | 15.1 μm |
| Prometa 4 | 150 μm^3^ |  |  |  |  | 15.1 μm |
|  |  |  |  |  |  |  |
| Metaphase 1 | 139 μm^3^ | 88 Mb/μm^3^ | 69 Mb/µm (Chr) | 0.68 ± 0.1 μm | 1.15 ± 0.2 μm | 12.68 μm |
| Metaphase 2 | 133.8 μm^3^ | 84 Mb/μm^3^ | 71 Mb/µm (Chr) | 0.66 ± 0.2 μm | 1.22 ± 0.2 μm | 14.54 μm |
| Metaphase 3 | 147.1 μm^3^ | 81 Mb/μm^3^ | 58 Mb/µm (Chr) | 0.69 ± 0.2 μm | 1.31 ± 0.3 μm | 13.69 μm |
| Metaphase 4 | 141.4 μm^3^ | ND | ND | ND | ND | ND |
| Metaphase  (Average) | 140 ± 4.8 μm^3^ | 84.33 ± 2.86 Mb/μm^3^ | 66 ± 5.7 Mb/μm  (Chr) | 0.68 ± 0.04 μm | 1.23 ± 0.1 μm | 14.16 μm |
|  |  |  |  |  |  |  |
| Anaphase 1 | 145 μm^3^ | 82 Mb/μm^3^ | 31 Mb/µm (Chd) | 0.68 ± 0.06 μm |  | 14 μm |
| Anaphase 2 | 142 μm^3^ | 73 Mb/μm^3^ | 32 Mb/µm (Chd) | 0.72 ± 0.08 μm |  | 17 μm |
| Anaphase 3 | 164 μm^3^ | 70 Mb/μm^3^ | 25 Mb/μm (Chd) | 0.61 ± 0.07 μm |  | 20.5 μm |
| Anaphase 4 | 196 μm^3^ | 55 Mb/μm^3^ | 27 Mb/μm (Chd) | 0.74 ± 0.07 μm |  | 21 μm |
|  |  |  |  |  |  |  |
| Telophase 1 | 228 μm^3^ |  |  | 1.06 ± 0.17 μm |  |  |
| Telophase 2 | 497 μm^3^ |  |  |  |  |  |
