# Supplementary material for: Near millimolar concentration of nucleosomes in mitotic chromosomes from late prometaphase into anaphase
Source: J Cell Biol. 2024 Aug 26;223(11):e202403165. doi: 10.1083/jcb.202403165 (PMC11346515; doi:10.1083/jcb.202403165)
Supplement: Table S2 — shows the difference between large and small chromosome distances from the spindle pole in anaphase. [file JCB_202403165_TableS2.docx]

Table S2. Difference between large and small chromosome distance from the spindle pole in anaphase.

|  | Spindle pole separation | Large Chromosomes  sK2P | Small Chromosomes  sK2P | % Difference between Large and Small Chromosomes |
| --- | --- | --- | --- | --- |
| Anaphase 1 | 14 μm | 6.84 ± 0.45 μm | 6.28 ± 0.45 μm | 8.18 |
| Anaphase 2 | 17 μm | 6.23 ± 0.64 μm | 5.61 ± 0.72 μm | 9.95 |
| Anaphase 3 | 20.5 μm | 6.20 ± 0.90 μm | 5.24 ± 0.83 μm | 15.48 |
| Anaphase 4 | 21 μm | 6.79 ± 0.94 μm | 5.54 ± 0.92 μm | 18.40 |

|  | Large Chromosomes  sK2K | Small Chromosomes  sK2K | % Difference between Large and Small Chromosomes |
| --- | --- | --- | --- |
| Anaphase 1 | 2.96 ± 0.37 μm | 2.49 ± 0.65 μm | -15.87 |
| Anaphase 2 | 7.01 ± 0.48 μm | 7.28 ± 0.77 μm | 3.85 |
| Anaphase 3 | 9.91 ± 0.4 μm | 10.64 ±0.74 μm | 7.36 |
| Anaphase 4 | 9.17 ± 0.73 μm | 10.72 ± 0.69 μm | 16.9 |
