# Supplementary material for: Near millimolar concentration of nucleosomes in mitotic chromosomes from late prometaphase into anaphase
Source: J Cell Biol. 2024 Aug 26;223(11):e202403165. doi: 10.1083/jcb.202403165 (PMC11346515; doi:10.1083/jcb.202403165)
Supplement: Table S3 — shows nucleosome volume calculation. [file JCB_202403165_TableS3.docx]

Nucleosome molarity calculations

T2T golden genome path = 3,054,815,472 bp

in mitosis (x 4) = 12,219,261,888

+ 1 extra XS 12 = 133,324,548 (x2) = 266,649,096

in mitosis (total) = 12,485,910,984 bp

**Chromosome total volume (metaphase)** = 140 ± 4.32 μm^3^

**DNA content density (average metaphase)** = 89.2 Mb/μm^3^

Assume that each nucleosome has 195 bp:

140 μm^3^ -> 89.2 Mb/μm^3^

457,359 nucleosomes/μm^3^

***OR***

135.68 - 144.32 μm^3^ -> 86.51 - 92.02 Mb/μm^3^

443,669 - 471,921 nucleosomes/μm^3^

Molarity:

457,359 x 1e15 x 1e6 / 6.02e23 = 457,359 x 0.00166 = 760 µM

***OR***

736.5 – 783.4 µM

Prometaphase 4 - 150 μm^3^ -> 83.24 Mb/μm^3^ = 426,869 nucleosomes/μm^3^ x .00166 = 708.6 µM

Metaphase 1 – 139 μm^3^ –> 89.8 Mb/μm^3^ = 460,650 nucleosomes/μm^3^ x .00166 = 764.7 µM

Metaphase 2 – 133.8 μm^3^ –> 93.3 Mb/μm^3^ = 478,552 nucleosomes/μm^3^ x .00166 = 794.4 µM

Metaphase 3 – 145.8 μm^3^ -> 85.6 Mb/μm^3^ = 439,165 nucleosomes/μm^3^ x .00166 = 729 µM

Metaphase 4 – 141.4 μm^3^ –> 88.3 Mb/μm^3^ = 452,831 nucleosomes/μm^3^ x .00166 = 751.7 µM

Anaphase 1 - 145 μm^3^ -> 86.11 Mb/μm^3^ = 441,588 nucleosomes/μm^3^ x .00166 = 733 µM

Anaphase 2 - 142 μm^3^ -> 87.93 Mb/μm^3^ = 450,917 nucleosomes/μm^3^ x .00166 = 748.5 µM

|  | O’Shea data (PMID: 28751582) | | | Rosen data  (PMID: **31543265**) | Our data |
| --- | --- | --- | --- | --- | --- |
|  | Euchromatin | Heterochromatin | Anaphase | Chromatin | Metaphase |
| CVC (chromatin volume concentration) | 12-21% | 37-52% | 40-55% |  | 32% |
| Nucleosomes per μm^3^ |  |  |  |  | 456,410 (calculated average) |
| Nucleosome + Linker Volume |  |  |  |  | 2186 nm^3^  (13 x13 x 13 nm) |
| Nucleosome molarity |  |  |  | 342 μM | 760 μM |
| Sample preparation | Chrom-EMT | Chrom-EMT | Chrom-EMT | Reconstituted chromatin and partial droplet FRAP | Chemical fixation, UA staining and resin embedding |

**Nucleosome core particle volume** = Diameter = 11 nm, Height = 5.5 nm

π r^2^ h = π (5.5)^2^ (5.5) = 522 nm^3^

**Nucleosome volume with DNA linker**

(195 bp / 146 bp) X 522 nm^3^ = 697 nm^3^ (nucleosome core corrected by 195bp/146bp)

**Nucleosome volume with DNA linker per μm^3^**

10^9^ / 697 = 14.34 X 10^5^ Nucleosomes/μm^3^

CVC Metaphase Chromosome = 457,359 / 14.34 X 10^5^ Nucleosomes/μm^3^ X 100

CVC Metaphase Chromosome = 32%

**DNA content density (average metaphase)** = 89.2 Mb/μm^3^

89 200 000 bp -> 1 μm^3^

195 bp -> X

X= 2186 nm^3^ = 13 X 13 X 13 nm
