# Supplementary figures and images for: Near millimolar concentration of nucleosomes in mitotic chromosomes from late prometaphase into anaphase
Source: J Cell Biol. 2024 Aug 26;223(11):e202403165. doi: 10.1083/jcb.202403165 (PMC11346515; doi:10.1083/jcb.202403165)

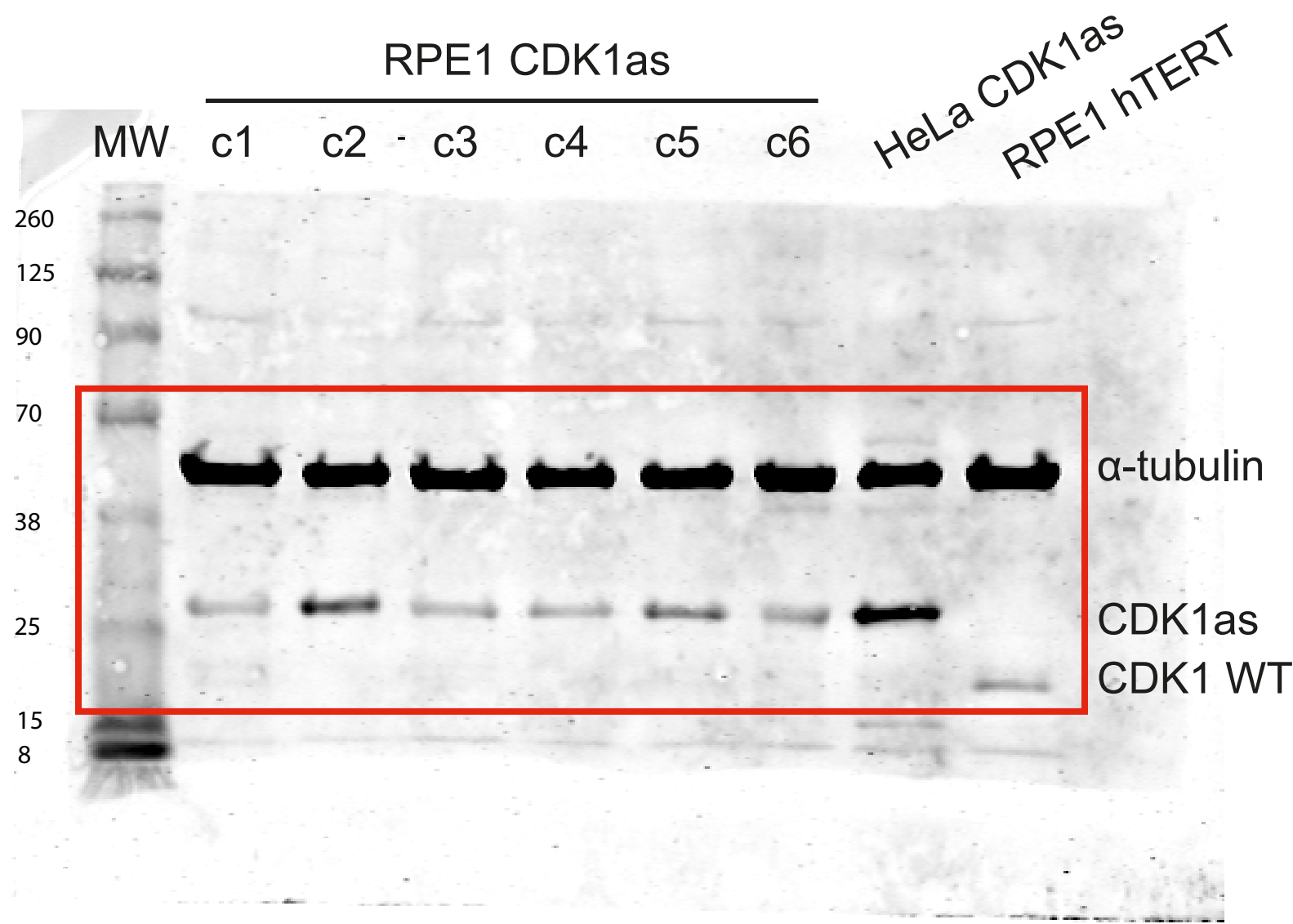

Supplement: SourceData FS1 — is the source file for Fig. S1. [file JCB_202403165_SourceDataFS1.pdf]
